# A Psychological Investigation of Selection Criteria for Learning Agents (Super Users) and Allocation of Responsibilities in the Implementation of Technological Change

**DOI:** 10.3389/fpsyg.2022.928217

**Published:** 2022-06-15

**Authors:** Jonas R. Vaag, Gunhild B. Sætren, Thomas H. Halvorsen, Stine D. Sørgård

**Affiliations:** ^1^Faculty of Social Sciences, Nord University, Levanger, Norway; ^2^Business School, Nord University, Stjørdal, Norway; ^3^Department of Psychology, Norwegian University of Science and Technology, Trondheim, Norway

**Keywords:** super user, super user selection, organizational learning, implementation, learning organization

## Abstract

**Purpose:**

In the introduction of new technologies into organizations, there has been an increasing trend to recruit and make use of the so-called “super users” to help ensure the future use of the technology in question. Little is known about the criteria that should ideally be considered in the selection of these super users, or about the best way to carve up the roles and responsibilities in this process between super users and middle management. In this study, we investigated (1) which criteria should be emphasized in the selection of super users, and (2) how middle management and super users understand and negotiate the responsibilities of their respective roles during implementation of technological change.

**Methods:**

We conducted 10 individual semi-structured interviews and used thematic analysis of this data set to identify selection criteria, roles, and responsibilities.

**Results:**

We found that the main selection criteria for super users should be: (1) availability and local knowledge, (2) technological skills, (3) pedagogical skills, and (4) proactiveness. The main roles and responsibilities that should be carved up between management and super users can be grouped into two overarching categories, each with several subcategories. Within the *Learning culture* category, the responsibilities are to (1) facilitate collective learning, (2) engage with criticism, and (3) promote collective sharing; and within the *Individual learning* category, to (4) facilitate individual learning, (5) provide instrumental support, and (6) provide emotional support.

**Discussion and Conclusion:**

Based on the findings, we propose a conceptual model of technological implementation and the construction of a culture of organizational learning, entitled ECo-System Of Learning in Organizations (ECSO-Learn); we additionally show how a learning agent (previously known as a super user) can be recruited to best fit into this model of long-term organizational learning.

## Introduction

Despite numerous attempts to identify the crucial factors involved in the process of successful technological change (e.g., Hong and Kim, 2002; Rizzuto and Reeves, 2007; Dwivedi et al., 2015), research shows that the majority of implementations of new technology are considered unsuccessful (Griffith et al., 1999; Hong and Kim, 2002; Woldesenbet and Klay, 2016). Many organizations tend to focus mostly on the technological aspects of the implementation of new computer systems (Kukafka et al., 2003; Aarts et al., 2004; Sætren et al., 2016), although the greatest challenges in this process are often linked to behavioral and organizational factors rather than technical ones (Lorenzi and Riley, 2000; Sætren and Laumann, 2017). There is a need for further research, from a system’s perspective, on which behavioral and organizational factors can affect the process of technological change.

The importance of developing employee competence is particularly relevant in the implementation of new computer systems because this often requires employees to learn to use new technology to be able to do their job (Shea and Belden, 2016). If the employees do not learn to use the system effectively, the new technology will fail to increase the organization’s productivity (Woldesenbet and Klay, 2016). In addition, research has shown that non-existent, poor, or inadequate training is directly correlated with low rates of adoption among employees, as well as unsuccessful implementation (Gagnon et al., 2012; Sætren et al., 2016). Therefore, in any assessment of the human and organizational factors that can contribute to a successful implementation of technological change, training of the employees is regarded as crucial (Wager et al., 2000; Åsand and Mørch, 2006; Keshavjee et al., 2006; Culler et al., 2009; McAlearney et al., 2012; Sitthidah and St-Maurice, 2016). Nevertheless, it turns out that training is often deprioritized and undervalued (McAlearney et al., 2012; Woldesenbet and Klay, 2016).

### Criteria for Selection of a Local Super User

Many organizations choose to use local super users to help other employees with the new system, to increase the chance of successful implementation (Kanter, 1984; O’Leary, 2000; Ash et al., 2003; Whitten and Bentley, 2007; Martinez et al., 2008; Crosson et al., 2011; Gagnon et al., 2012; Sitthidah and St-Maurice, 2016). Super users are usually regular employees who receive additional training in the use of a new computer system to be introduced at the workplace, so that they can provide first-line technical support and training to their colleagues locally (Åsand and Mørch, 2006; Boffa and Pawola, 2006). Although the use of super users is a widespread strategy in the workplace, we lack sufficient understanding of the preferred roles and behaviors of these super users (Shaw et al., 2012; Shea and Belden, 2016; Woldesenbet and Klay, 2016), and there is a lack of psychological research on this topic. The lack of clarity in the literature on super users further prevents the development of research-based approaches to identify, support, and assess the impact of super users (Shea and Belden, 2016).

### Super Users – Leaders, Managers, or Change Agents?

Leadership and management are key factors in the processes associated with all forms of change (Gill, 2002; Herold et al., 2008), and effective leadership has been identified as a key factor in the success of change initiatives (Kotter, 1995; Todnem, 2005; Hornstein, 2015). Leadership can be understood as a set of functions and roles that must be fulfilled to address all important aspects of the workplace and processes occurring within it (Mintzberg, 1990), and the ability to lead employees in a change process is often considered to be one of a leader’s most important tasks (Burnes, 2004; Todnem, 2005). Middle management often plays a central and important role in change processes (Rouleau, 2005; Bryant and Stensaker, 2011), but many middle managers are often stuck handling administrative tasks and addressing the requirements and needs of different parts of the organization (Johansen and Hawes, 2016; Chen et al., 2017). Furthermore, middle managers often implement change processes while maintaining the continuity of the organization’s daily work. A single leader does not necessarily possess all the leadership skills needed to promote optimal efficiency in their teams and groups (Pearce and Manz, 2005). Another crucial factor is the quality of the relationship between the leader or manager and their employees (Anand et al., 2011), which is partly dependent on the relational competencies of the leader, such as altruistic leadership (Salas-Vallina and Alegre, 2018). Although management is most often thought of as vertically organized and executed by a formal and designated leader, leadership can also be understood as a collective phenomenon in which management tasks are distributed among several people in a group (Pearce and Sims, 2002; Morgeson et al., 2010). This form of shared management has also been shown to have a greater impact than traditional vertical management on efficiency in groups (Pearce and Sims, 2002).

For these reasons, even though middle managers often act as change agents who manage and make changes to their respective departments in a change process, employees can also play a central role as change agents (Lunenburg, 2010). In the introduction and implementation of new technology, it is common to involve super users as part of the process (Yuan et al., 2015; Shea and Belden, 2016). In the literature on this topic, several studies argue that super users exert a strong influence on whether the implementation of a new technology is successful (Shea and Belden, 2016), and that super users’ contributions can be essential in technological change (Halbesleben et al., 2009; Shea and Belden, 2016).

### Research Questions

There is a lack of research on the psychological aspects of the selection of super users and the roles and responsibilities of super users and middle management in processes of technological change. Thus, in this study, we investigated (1) which criteria should be emphasized in the selection (and training) of super users, and (2) how middle management and super users understand and negotiate the responsibilities of their respective roles during the implementation of technological change.

## Materials and Methods

To identify the optimal criteria for the selection of super users and to investigate how management and super users understand and clarify their roles in the implementation of technological change, we used an inductive explorative qualitative approach. We chose to collect data through semi-structured interviews of employers, management staff, and super users in an organization that recently had implemented new technology. The project was reported to the Norwegian Centre for Research Data (NSD).

### Researchers’ Background

The researchers’ background is in organizational psychology. Two of the researchers each hold a Ph.D. and an associate professorship in psychology and have several years of experience in conducting and teaching qualitative research, as well as working on organizational change and implementation of new technology. The other two researchers each hold an M.Sc. in organizational psychology and work daily with organizational management and change processes in praxis.

### Context

The organization in which the interviews were conducted was a Norwegian municipality with more than 10,000 employees in roles ranging from basic office jobs to those involving close contact with the municipality’s inhabitants, such as health care and fire services. There are 356 municipalities in Norway, each of which is organized similarly. A new technological system for mail, document sharing, meetings, and so forth was implemented for all employees, throughout the entire governmental organization.

### Recruitment Process

In total, 10 informants were recruited to participate in the study. A predefined criterion was that each informant must work in an organizational unit that used the new technological tool daily and must either have access to a super user or be a super user. We wanted to recruit participants from different positions within the units and different units, to shed light on the process of technological change from different perspectives. Participants in the study volunteered *via* a registration form which was posted on the organization’s intranet. Five men and five women who volunteered to participate were selected as informants based on their relevance. Thus, individual semi-structured interviews were conducted with 10 informants from three different units in the organization: the project leader of the technological implementation; six employees (of whom three had carried out the role of a super user); and three middle managers.

### Interviews and Interview Guide

Data were collected using individual semi-structured interviews (Kvale, 1996). All interviews were conducted at the workplace of the informants without any interruptions and without the possibility of others overhearing the conversation. The objective in this choice of location was to secure a well-known environment for the informants as well as to respect the demands of their work and avoid taking too much of their time. Each interview lasted approximately 1 h; all were recorded and transcribed.

An interview guide was constructed; this included questions covering aspects of the nature of the technological change itself and the change process. The initial part of the interview concerned a normal working day for the informant. This was included to generate a more comfortable atmosphere and allow the informant to talk about a non-controversial and safe topic, while also providing important contextual information. To explore our themes of interest, we additionally included interview sections with questions concerning the role of super users, the demands facing them, the resources required to function effectively as a super user, and the expectations of both the super user role and the role of management in technological change. This section of the interview included questions such as “Can you describe your role as a super user?” and “Why did you choose to make use of a super user in this project?,” depending on the role of the informant being interviewed. In addition, questions were included about how the new technological system worked and about the informant’s reflections on the consequences of its implementation in the organization. We also asked open-ended questions concerning the informant’s role as a leader in the change process, for example, “What do you see as your most important role as a leader of change processes?”

After conducting six interviews, we found it necessary to revise our interview guide, following the themes that emerged through the initial analysis. Following these themes, we added more specific (but still open-ended) questions relating to the characteristics required of super users, and to the associated roles and responsibilities, and the need for clarification of the allocation of these roles between super users and management: for example, we included the question: “How would you describe the roles carried out by you as a leader and the super user in praxis?” In addition, themes including the context of the informant’s work, aspects of their use of technology, and consequences of the change were included to allow us to obtain a broad understanding of the implementation process. An example of an open question was “How would you evaluate the training you received on the new technology?,” and follow-up questions to this could be, for instance, “How did you perceive this training?” or “What did the training consist of?” All interviews ended with a question about whether the informant had anything to add or felt that there was anything we had forgotten to ask about.

### Analysis

Thematic analysis (Braun and Clarke, 2006) was used to analyze the data, and Ose’s (2016) approach was employed to organize our analysis of the written material into categories. Thematic analysis is a flexible approach for the analysis of themes identified in interviews based on a constructive and inductive approach. In addition to this analysis, we conducted a literature review in parallel with the data collection and analysis, in order to obtain an in-depth understanding as part of the analysis process. Although the analysis method consisted of six steps, the process of analysis was flexible and dynamic, as we shifted back and forth between these steps to make necessary changes as per the research question and purpose of the study. Despite the adoption of an open approach with subjective constructions of the relevant themes, the process of analysis also involved critical thinking and the drawing of comparisons to assess the findings appropriately. During the thematic analysis phase, multiple researchers conducted their analyses in a parallel analysis process. Their individual findings were compared and coordinated to produce a final analysis and construct a model (Braun and Clarke, 2019, 2022).

**FIGURE 1:**
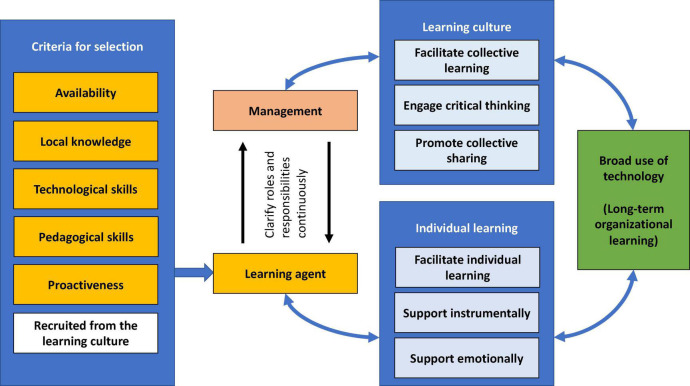
The process of selecting a super user (learning agent) and the allocation of roles and responsibilities between the agent and management to ensure broad use of technology.

The first step was to become familiar with the data. This was achieved by conducting the interviews and transcribing them before thoroughly reading through the transcripts. During this process, ideas relating to initial categories and patterns emerged, and notes were taken. The second phase consisted of initial coding, in which the material was sorted into codes. Examples of codes from this phase were “The style of management affected the unit” and “Super users made their own teaching handbook.” In total, more than 300 codes were identified during this phase. In the third phase, the process of organizing the data into meaningful categories was initiated. This was the point at which we began to discover the underlying themes by applying more in-depth analysis. During this phase, we discovered connections between elements that had not necessarily been obvious from the start of the process (Braun and Clarke, 2022), and these connections formed part of the process of combining the initial codes into themes (Braun and Clarke, 2006). During phase four, the themes were revised. This phase consisted of two sub-phases: in the first, we examined the coded material to ensure that connections were present within the themes; and in the second, we confirmed that the themes identified were congruent with the remainder of the data. As an example of the development of codes, the previously mentioned code relating to super users making their handbooks was included in this fourth phase in the theme “Super users designed courses for the end users.” During the fifth phase, the researchers identified the essence of each theme and what it represented, as well as identifying which were major themes and which were sub-themes. For instance, within the theme of preferred super user characteristics, the “proactiveness” criterion, which was subsequently incorporated into our ECo-System Of Learning in Organizations (ECSO)-Learn model (Figure 1), was developed at this stage. The objective at this point was to ensure that the findings were constructed on a solid foundation and to verify their relevance to the research question. Throughout this entire process, quotations relating to the themes were collected and examined, and memos were written. The process of theme development was inductive, meaning that it was guided by the interview material that was collected rather than being theory driven. The sixth and final phase of thematic analysis was to write down the findings in the form of a scientific article (Braun and Clarke, 2006).

### Validity

Several approaches to establishing the validity of qualitative research findings are available (e.g., Kvale, 1996; Elliott et al., 1999; Yardley, 2000). For this study, we chose to apply Yardley’s (2000) four criteria, with an emphasis on providing transparency for the reader within the scientific context on the basis of these criteria. Yardley’s (2000) first principle is sensitivity to context. This refers to the importance of providing theoretical, scientific, and social context. For this reason, we have presented a relevant theoretical framework in the introduction, described our professional backgrounds, and explained the work context of the organization and informants. Furthermore, our explanation of the data collection and analysis includes a description of the process, while the results are reported through both quotations from the data and presentation of our interpretations, in accordance with the second criterion: thoroughness of the data collection, analysis, and reporting. The third criterion relates to commitment, rigor, and coherence, and includes the requirement to explain the authors’ degree of engagement with the research context, their methodological skills, how the data collection was carried out, and the process of analysis. We have therefore described our relevant background; additionally, every stage of the research, from planning to writing, was conducted by two or more members of the author group. Furthermore, we have described the processes employed and remained close to the data throughout collection and analysis. Thus, we regard this research as transparent. The fourth and final criterion proposed by Yardley (2000) is impact and importance; these facets of the study are analyzed in section “Discussion.”

### Ethics

This study was approved by Sikt (the Norwegian Agency for Shared Services in Education and Research, known as NSD before 1 January 2022) and conducted according to this organization’s ethical guidelines and regulations, which include de-identification of the interviewees, voluntary participation, and appropriate handling of the data. All participation was voluntary, and informed consent was obtained from each informant.

## Results

First, we sequentially presented the themes that emerged from the analysis (Table 1), categorized as either *selection criteria* (relating to the selection of the super user) or *role allocation* (relating to the allocation and negotiation of responsibilities between managers and super users). An important finding was that these two processes are closely linked and that the process of role allocation and clarification between a super user and a manager is a continuous one lasting throughout the learning process. Subsequently, we described the different themes and sub-themes that are used in the construction of a conceptual model of our results; the model itself is presented in section “Discussion,” along with further analysis of the overarching implications of the findings.

**TABLE 1:** Themes identified in the thematic analysis.

Selection criteria	Role allocation
● Availability and knowledge of local norms ● Technological skills ● Pedagogical skills ○ Communication skills ○ Ability to provide customized training and help ● Proactive job-crafting ○ Initiative-taking ○ Interest, innovativeness, and willingness to learn	Learning culture ● Facilitate collective learning ● Engage with criticism ● Promote collective sharing Individual learning ● Facilitate individual learning ● Provide instrumental support ● Provide emotional support

### Criteria for Super User Selection

#### Selection Criterion 1: Availability and Local Knowledge

The first criterion for super user selection is that the super user must be present and available to employees to successfully support and facilitate their learning. Furthermore, the informants pointed out that the super user must be employed by the local department. In this way, employees have a person who is always available to help at any time.

*“I do not see for myself how to do it differently. Because then you always have one on the unit. If you were to hire a person then that person would have to come here and instruct us, so it would always have been that you would have to contact that person, and then production would have stopped until that person had arrived then. But since we have a **super user on the unit** who is here daily, the super user is **always available**.”*

*“Good competence, pedagogical skills, and* ***presence***. *I will point these out as core factors for it to work well. (…) there are many people who are out of office. And if one of them had been the super user, then I do not think it would have worked properly.”*

The above quotations indicate that other solutions would adversely affect the efficiency of the employees’ work. In addition, the super user should be a person who, due to their working conditions, is physically present at the department, and who can actively express their availability and take the time to provide help when needed. *“If there is anything, it is no problem to come to me to ask for anything*… *it is about being available and saying yes.”*

#### Selection Criterion 2: Technological Skills

Technological skills are, unsurprisingly, highlighted as an important criterion in the selection of a super user. However, the informants varied with regard to the level of knowledge and skills that they believed to be required. The majority pointed out that the super user must have a high degree of expertise in the system that is to be implemented, and that they must have a full understanding of all the different parts of the system:

*“The person needs to understand what it is that he or she is a super user of. I think it’s really important (…) You have to have good knowledge of the tools. Simply. The whole toolbox*.”

Many of the super users, however, pointed out that the training they received was not sufficient to enable them to do a good job as a super user. Therefore, if an organization is to avoid investing in extensive training for super users, the choice of super user must be based on who has pre-existing competence with the system, sufficient time to learn about the system on their own and interest in doing so, and a special interest in this area and technological skills in general.

*“The way I see it, it’s hard for me to see who could take the job without it reducing productivity in other work. Because people have pretty much on their table already, and being a super user requires a lot of expertise. And it may require more expertise than what you got through the training.”*

*“At least I hope that those who have become super users had a pretty good basic understanding before they were chosen. Because without that basic understanding, it will become difficult.”*

Despite this perspective, some informants also reported considering it less important to have previous knowledge of the system. Under this view, the super user will be able to do an equally good job as long as they have an interest in and preference for technology in general: *“You should know how to turn on a PC, to put it nicely. You should have some interest in IT and be fond of using technology.”* Furthermore, some argued that the most important thing is to know what opportunities lie in the system, where these opportunities can be found, and whom to contact for help. Here, the focus is less on technological skills, and more on being engaged, educational, and service-oriented in the role.

#### Selection Criterion 3: Pedagogical Skills

All of the informants mentioned pedagogical skills as a prerequisite. This theme was divided into two sub-themes, one concerning pedagogical communication skills, and the other concerning the ability to provide customized forms of training to serve the individual needs of employees. A more detailed description of each of these sub-themes follows, with illustrative quotations.

##### Communication Skills

Pedagogical skills were characterized, among other things, as good communication skills, with a focus on the ability to explain things in different ways to different employees.

*“You have to have some pedagogical skills, so you can teach others.”*

*“I think it’s nice if you’re good at different forms of exchanging knowledge…you can explain things in more ways than one, because people do not learn things the same way.”*

These statements emphasize the fact that people learn in different ways, and that the super user must have the ability to adapt accordingly. Regarding communication, some informants pointed out the importance of training and help is provided in such a way as to have a calming effect on employees.

*“It is important to be able to convey and to show things in an understandable way. The super user should not just rapidly show the solution. You have to try it for yourself in a stress-free way.*… *and say ‘If there is anything, it is no problem to come to me to ask about anything.”’*

Several of the informants stressed that pedagogical skills were one of the most important factors and suggested that training in pedagogical skills should have been given greater emphasis in the training of super users.

*“And then there are certain prerequisites when it comes to pedagogical qualities and patience that must surely be in place. (…) And then there is the dissemination of knowledge.”*

The informants representing the super users also reported that their experience of the corresponding training was that it favored technological competencies and gave them little time to work on the pedagogical aspect of their role as a super user.

*“We only received training in the tools themselves*…”

*“… we got a very simple introduction to the technological system, and the training was _very_ simple. But what I see as a problem is that there were a lot of people who became super users. But there was no such thing as an agenda on how we should present it to the employees. No such methods. It was like just: ‘Here you get the training, and then go out on the unit and…’ But not HOW should I present it. What should I focus on? So it was very basic. So I had to find out for myself then, how should I get people to (learn), what should I teach them, what is it important to focus on?”*

*“And then this fear of using new technology*… *we have not received any training in handling that*… *the pedagogical way of implementing it…”*

##### Customization of Training and Help

All of the informants pointed out that customizing the help and training provided to colleagues was one of the most important skills of the super user. The super user had to be able to adopt both training methods and the content of the help provided to individuals’ needs and their levels of technological skill. To be able to adopt the training they provide to the relevant employees, super users should be recruited from the local unit, as this means they will have local knowledge; this was also highlighted in criterion 1, as working in the relevant department means that the super user possesses useful information about routines, the department’s employees, the various work tasks, and the like. With this knowledge, local super users can adopt the help and training they provide to the relevant department to a greater extent than an external super user would be.

*“A unit is so complex that it’s not possible to just get in there and not quite understand what the work processes are like. None of the units are alike here, no two units are alike.”*

*“So we had to find a model that made the training go well.*… *so we fully agreed that we must use the employees. Because they know how the shoe fits and what is important to the department. They know what the work tasks are in their unit and so on. I think that if we had followed the slightly more traditional way where we had demanded that the supplier should provide 20 men, then there would have been 20 people who did not know the units, who knew nothing about them, had no idea about the work tasks, but who just slavishly presented the tools without knowing anything about how they could use them to solve the tasks.”*

#### Selection Criterion 4: Proactive Job-Crafting

The ideal super user should be proactive in the way they work. Proactivity in this context is about being a little “ahead,” and contributing in an active way to other employees’ learning processes. In addition to a desire to help others, this level of commitment involves an interest in learning the new technology and finding better solutions. The theme is illustrated by quotations that are categorized under the sub-themes of “initiative-taking” and “interest, innovativeness, and willingness to learn.”

##### Initiative-Taking

Being committed to the role and inclined to take the initiative were described as important characteristics of a good super user. This theme is about the super user working proactively and taking the initiative in adopting various measures that can make the learning process easier for employees. When asked about how the super user should work, many informants answered that they valued this type of unsolicited training: *“Come up with many tips and tricks, maybe be a little on edge, run some training internally, yes, a bit like that unsolicited, maybe. Be a little on edge”*; *“Maybe come up with some tips and tricks for us without us asking for it…”* Several of the informants pointed out that there is a lot to learn when a new computer system is implemented, and that it can therefore be difficult for employees to discover all the possibilities that the system offers. For this reason, it is important that the super user has an overview of the various functions, and actively shares information about the possibilities inherent in the system.

*“As I said, the [employees] do not currently use anything other than e-mail then…But it is about making them use a little more of the technology then, the fact that you can make them see the opportunities there. That you as a super user can show that there are many possibilities.”*

Several informants also talked about a sense of responsibility and commitment in the role of super user. One of the informants said that 1 year after the system was implemented, she was still setting up internal courses to demonstrate new and useful functions that appeared in the system, in addition to providing training for new employees.

##### Interest, Innovativeness, and Willingness to Learn

The informants reported that their experience indicated the importance of the super user’s ability to show the employees what opportunities lie in the system. For this reason, a super user’s interest in identifying better ways to accomplish work tasks using technological aids is also considered an important factor. This sub-theme is therefore about being curious and innovative and seeing opportunities rather than challenges in new technology. The informants explained that the super users must be committed and willing to familiarize themselves with the system on their own, in addition to the training they receive: *“You must be willing to learn yourself, I think*.” Additionally, the super user needs to be prepared for questions that may come from the employees.

*“That you understand things quickly. And that you can understand more than exactly what you are told. It has been the idea that when I meet with the unit and they ask about things that I have not been trained in myself, then I have tested it, or found out for myself because that*… *It is not so difficult to train me in technical computer programs. It actually goes pretty fast! That you can…that you have an interest in teaching yourself more, but also others.”*

In addition to this interest in learning the system for themselves, a super user’s commitment must also involve a desire to help others. The following quotation illustrates how a super user who is curious and eager to learn can explore the system and discover useful features that can make their own and others’ work tasks easier:

*“What I see that is useful is a lot of such extensions then, which we have not received any special training in. Which extensions are we going to use…and things like that, so there is a lot there that I have found myself then. And so I have told other employees that it may be a good idea to try them, such as an extension that allows you to save everything you copy, so that you get a list of everything that has been copied. (….) I think they could have used me more really…Just figuring out how they can use the system, not just coming to me when they have a problem, but maybe also with how they can use it.”*

The informant’s statement illustrates how the super user’s initiative in training the employees functioned as a good supplement to the training the employees were offered by the workplace, which focused more on the technical aspects of the system than on useful functions. As mentioned above, both employees and super users reported believing that it is important to receive training in the possibilities offered by the system, and not just in how it works. Furthermore, this sub-theme suggests that committed and enterprising super users can contribute to making colleagues’ everyday work more effective through their learning and knowledge sharing.

*“Hehe, I am very much a ‘yes-person.’ I say yes to everything. Now I am also a super user in a new accounting system…When you get new systems, it’s fun to be able to influence [things], I get to be involved in influencing both myself and others’ everyday work. When I then get to participate in design and say ‘here is something missing,’ ‘here is the potential,’ ‘this is good…’ I was saying, if one is to be a little selfish and think that if someone is going to shape my workday then I would like to help shape how it should be.”*

One of the informants compared an ideal super user with his views on the ideal employee for an organization more generally.

*“Most important? Eh, ‘positive,’ ‘solution-oriented,’ ‘interested in technology,’ ‘collaborative.’ This is the employee that the organization wants everyone to be, and that many are. Whoever is positive about change, wants to try something new, is innovative, sees that this opens up smart opportunities to make things easier, smarter. The organization has 15,000 employees, and we cannot hire new people every time there exist new tasks to be done.”*

The focus on innovation, curiosity, and the ability to see technological possibilities for simplifying work tasks may indicate that the informants also perceive willingness to learn as an important characteristic of a super user.

### Roles and Responsibilities of the Manager and Super User

In the analysis of allocation of the roles and responsibilities between management and super users, a clear trend emerged indicating the importance of the fact that the super user is part of an organizational learning culture, and that the organization’s cultural maturity is an important factor in how the super user should work with the existing learning culture within the organization. It also became clear that how roles and responsibilities are shared between the manager and super user is continuously undergoing clarification and re-negotiation.

The theme of continuous development of a learning culture consisted of three sub-themes: “facilitate collective learning,” “engage with criticism,” and “promote collective sharing.”

#### Role Responsibility 1: Facilitate Collective Learning

Several informants emphasized the importance of a culture that is characterized by a curious attitude toward learning. It appears that both the manager and the super user play both a formal and an informal role in developing this learning culture. The willingness of the manager or super user themselves to engage in learning is important for their ability to influence the attitudes of other employees:

*“I see it in those units that have particularly super positive leaders, where the management of the unit takes the lead and show how they do it and that we fix this. And give time for learning to the employees. ‘I understand that this takes time. Of course, you should have time to familiarize yourself with this. Then it will be good.’ But where you might have someone who says that ‘we cannot spend time on this,’ then it becomes the starting point for not spending time to understand and see opportunities, then one begins to cultivate this negativity*…”

It appeared to be important that the manager promotes a realistic approach toward learning and also understands that doing so takes time and resources. A manager who does not facilitate learning, and who does not allocate time and resources for learning to take place, will in turn be responsible for a deterioration of the collective learning culture:

*“‘Oh, this is what we have been waiting for.’ That is what many have said. Many of the positive workers have been enthusiastic about the new technology. It’s a little contagious. We also see that in the units where things are a bit negative, it is actually negative all over the place. Because it is such a cultural negativity, if I may call it that…So one can ask the question: are they then pushed down by the positivity so that they do not dare to say that this is hopeless? Or is it actually the case that the culture is like that, that ‘we fix this, and we help each other with it?’ I think it’s the last thing.”*

Several of the informants also pointed out that units vary in terms of their cultures in relation to being innovative and taking advantage of new opportunities. The quality of the learning culture contributed to how the tool was received by the employees. Several of the leaders described experiencing a positive culture in their own unit:

*“So yes, I want to say that there is a kind of culture on the unit here…Because we like to say that we are a little forward-thinking and a little innovative.”*

*“… We try to play with what is the advantage of this, not on what we lose…We try to play with the advantage of working together on things.”*

#### Role Responsibility 2: Engage With Criticism

Engaging with employees to encourage them to participate in the learning process and to provide opinions, perspectives, and knowledge also became apparent as an important factor in the descriptions given by the informants. An important role for the manager is to make room for such conversations and differences in experiences and opinions.

*“I live with the perception that I am not someone who knows everything here. The experts are my employees. I have to make it easy for them to figure things out as best I can. And they are much better than me, so I see no reason for me to think all the thoughts alone. But giving them the opportunity to explore further. Bringing them together in a team, discussing if I should hear things, hearing them when they come to me.”*

#### Role Responsibility 3: Promote Collective Sharing

The middle managers and super users reported feeling a sense of responsibility in facilitating the extent to which the possibilities of the new technology were exploited. The informants pointed to the sharing of knowledge, both within and across the departments, as important in reaping the benefits of the new tool. They pointed out the importance of facilitating a sharing culture in which experiences with new functions of the tool were made available to other employees. Knowledge sharing was relevant because there were several functions and updates to the tool that it had not been possible to review during training. Several of the informants reported that knowledge sharing was an advantage because it made the work more efficient and provided better quality collaboration, and new forms of collaboration, both within and across departments:

*“(…) So I think different things are used from unit to unit. But at the same time, we are encouraged to have a sharing culture, and it is important that if we discover any new opportunities, we are asked to share.”*

The role of middle managers was often to encourage employees to share their experiences and knowledge, or to seek help if there were specific things they were wondering about. There was also a perception that both managers and employees had a joint responsibility to stay up to date on relevant information. At the same time, a sharing culture was also incorporated before the introduction of the new tool, meaning that practice in relation to the new platform continued a practice that had already been incorporated and facilitated by the leader:

*“I think the best thing we can do is to let employees find out things as they happen. That you discuss challenges, and come up with good solutions…So that way, I cannot say that I have had any special role, other than that I have encouraged [people] to just share. It is and quite natural when we have a group to share things with. Then we share a lot.”*

*“I live with the perception that I am not someone who knows everything here. The experts are my employees. I have to make it easy for them to figure things out as best I can. And they are much better than me, so I see no reason for me to think all the thoughts alone. But giving them the opportunity to explore further. Bringing them together in a team, discussing if I should hear things, hearing them when they come to me.”*

*“We have good experience of sharing information with each other. Because we have different interests and fields of knowledge, and then it’s about making the most of it for the benefit of all.”*

#### Role Responsibility 4: Facilitate Individual Learning

Training consisted, for instance, of key courses for employees to attend, training materials that were made available online, and training sessions held by the super users within their departments. The middle managers and the super users facilitated the learning process among employees. Middle managers could send employees on courses or ask them to go through the training material online, ask for extra help and resources from the project group for training within the department, or request that the super user hold internal training sessions. The super users, in contrast, were often the ones who underwent the actual training and technical review. The informants reported that various forms of training were carried out, and also addressed aspects of these that were particularly relevant to their department:

*“I encourage everyone to attend courses. Those courses were primarily for managers, but I think 2/3 of those who are here went on one. I sent them on that course. Simply so that they would have enough foundation to use it right away. So in that way, I think I have had influence, but at the same time the unit is not very backwards. They work digitally, and we work with a PC, it is our most important tool…”*

The super users were given a high degree of freedom to design the training provided in their own unit.

*“They were given free rein to do that, there is nothing we can do about it, but we knew that the units had a super user. As the unit leader had approved it, and the unit leader had said that this should be ours. And then there were unit leaders who had given them room to call in for courses, they had set up several courses, they simply took in employees for courses at the unit. Other units that were small, where they ran around when people got the accounts and helped them to their seats instead. True, there are many ways to do this.”*

*“Some already had people who were going to say that there is a difference in the demographics of the units in the municipality. We have units that have a lot of people well up in years who may find this difficult, and who have not been so much inside this type of tool, and then we have units that have a lot of young people who then may be more used to it, and may have always used these tools. Always had a Google account and a Gmail. So there was a difference between units and then. But there they had free rein, so there was a lot of variation in how they solved this.”*

Several of the informants said that it was largely up to the super users to take the initiative on how the training should take place. The provision of effective training was therefore dependent on the super user’s ability to see what needs and challenges there were in their own department. The super user also had to influence to make this happen in their own department. There was therefore often a collaboration between the super user and the middle manager to achieve this:

*“The training I ran on my unit was something I initiated myself. I brought it up with the unit manager that this is something I want to do, and she said ‘run on,’ so then I called in for training and then I ran training on the things I felt they needed, and then they came back with questions, and then I said I was going to run a course on what they were wondering about next week. That’s how it rolls and goes. So it was something I did on my own unit because I felt that what I learned in the tutorial might not be something my unit could handle. So I tried to close the knowledge gaps we had on that unit here.”*

*“And then this fear of using new technology, we have not received any training in that. That is, how, like, the pedagogical way of implementing it. There was no focus. So that was how I had to address it on my own, and find out.”*

#### Role Responsibility 5: Provide Instrumental Support

One way to address the needs of employees in the learning process was to deliver support. Instrumental support, in the form of help with technical problems and challenges with the tool, was a form of support that was emphasized as being important. Productivity may drop if employees experience problems they are unable to solve on their own.

*“It is finding solutions for people when there is a problem. And giving tips on ways to work, and so on.”*

*“No, I think that’s right, there were quite a few questions in that introductory phase. So that the [… *technology*…] was earmarked for a certain amount of its working time to guide co-workers in training and mentoring, it is certainly important…”*

Both middle managers and super users had a role to play in supporting employees in this process, but the informants pointed out that the super user was often closest to the challenges the employees faced and the one who had the greatest technical expertise. It was also particularly important that the super user was present and available in the department when these challenges arose. The informants also emphasized the importance of having a specific person they could contact, who knew about the work in the department and who could easily understand the challenges any given employee faced:

*“Yes, I think as far as it has been very good, it is a person we have gone to when we have had questions and we have received good answers.”*

*“I do not see how it could be done differently. Because then you always have one on the unit. If you were to hire a person, that person would have to come here and be instructed, then it would always be the case that you had to contact that person, and then the work would stop all the time until that person had come then. But since we have a super user on the unit who is here on a daily basis, you are always available.”*

Although super users were happy to help solve problems, they often had to seek help from other parts of the organization as well. The super users thus often acted as a link to the source of the help needed.

#### Role Responsibility 6: Provide Emotional Support

The informants revealed that the super user had the opportunity to influence employees’ willingness to learn when the new technology arrived. However, at the same time, that influence could be limited by the current organizational culture: it was easier to influence the employees in a unit where the organizational culture had already adopted an essentially positive attitude toward the system. One informant emphasized the challenge that a super user will experience in their work on a unit with a lot of skepticism:

*“Yes, it will probably be able to do that. But I think he has a much harder job. I see that we have very powerful super users who have managed to turn many around. It’s a very important job they have. But they can quickly end up working in a headwind if they do not receive any support on the unit.”*

In connection with uncertainty and resistance, several of the informants pointed out the importance of the super user’s ability to maintain a positive and supportive attitude, although they receive little support themselves. The informants described this as being patient and having stamina. Furthermore, several informants talked about what kind of influence a super user can exert by actively focusing on supportive learning of the new system, and by maintaining a positive outlook in the face of skepticism.

*“In our finance and accounting department, we had a pilot phase, and there was a super user who when someone said that this does not work, he [the super user] focused on showing how it could work. ‘Look here now, see how easy.’ That’s the way to turn things around. So the skilled super users who have turned people around, they have simply said, ‘I hear what you’re saying, but look here.’ And then they have set good examples, contributed, shared. So that’s what helps turn things around.”*

## Discussion

In this qualitative study, we investigated (1) which criteria should be emphasized in the selection of super users, and (2) how middle management and super users understand and negotiate the responsibilities of their respective roles during the implementation of technological change. In our analysis, we found that several relevant characteristics and skills should be taken into consideration in the selection of a super user, including local knowledge and pedagogical skills. We also found that the roles and responsibilities of management staff and super users could be grouped under two main headings of fostering individual learning and fostering a collective learning culture. This finding is in accordance with a socio-cultural perspective on learning, and with how Lave (1991) and Wenger (1998) describe how learning is situated, and how it occurs on both individual and collective levels through interactions in, what they describe as, communities of practice.

Based on our findings, we proposed a conceptual model (Figure 1) of the process of selecting a super user, whom we now refer to as a *learning agent*, and clarify the allocation of roles and responsibilities between this learning agent and management to ensure that new technology is implemented broadly and comes into use widely, as well as the process of building a long-term culture of organizational learning.

The basic premise of this model is to combine a systems approach with the perspective of organizational learning. A systems approach is based on considering the organization from a systematic rather than an individual perspective (Leveson, 2020). The adoption of such an approach encourages customization of the system to make it as reliable as possible in enabling individuals and groups to carry out their jobs in an optimal way. Through optimization of the daily working context for the humans involved and the tasks they are to perform, the organization will run smoothly.

For these reasons, management must provide the relevant tools and coordinate the relevant environmental factors in such a way as to enable employees to focus on their core tasks. This includes social structures as well as instrumental and physical factors (Leveson, 2020). During a change process, this becomes particularly important, as changes always will consume additional resources and change is regarded as a stress factor. One of the most important factors in enabling an organization to maintain productivity during this time of stress is the provision of extra resources to employees, such as a local super user. Our results show that the super user (whom we now refer to as the learning agent) is not only responsible for being an expert in the system or technology that is undergoing implementation within the organization; a local learning agent should also have expertise in learning and in process and knowledge management, with extended roles that should be clarified in consultation with the local manager. This is somewhat in line with Rizoto-Vidala-Pesoa and Kuzņecova’s (2017) suggestion that a super user should have a permanent process management role in their organization. However, our argument is specifically that learning agents should be involved in the facilitation of their organization’s learning culture.

**FIGURE 2:**
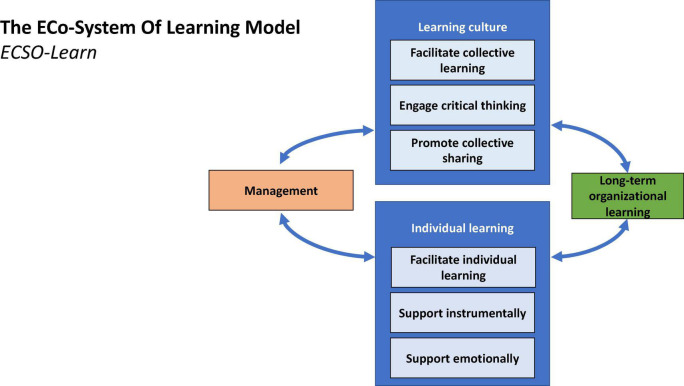
Proposed model of continuous and long-term organizational learning.

For a social working environment to support a culture of learning, it must encourage its inhabitants to engage in critical thinking. The data presented in the current study have provided examples of embracing critical thinking, as the informants were open to suggestions that would enable them to exploit the local knowledge of learning agents. Critical thinking and open discussion in an environment of psychological safety (Edmondson, 1999) are important features of any highly reliable, resilient organization with a strong culture of learning (Weick and Sutcliffe, 2015; Leveson, 2020). To perceive new opportunities and identify new ways to optimize work processes and the social aspect of the working environment, it is very important to adopt a critical outlook and embrace critical thinking. Thus, organizations should tailor their systems to include critical voices. This could, for instance, be achieved by rewarding individuals who can express disagreement in a respectful matter and demonstrating an inclusive approach to skepticism by bringing critical voices to the table and discussing criticism openly. For this reason, social interaction is an important factor in an organizational learning culture or a learning organization (Figure 2).

### The ECo-System of Learning Model in Relation to Organizational Learning

To explain how the model presented above relates to organizational learning, it is necessary to elaborate on the core principles on which it is based, which incorporate a certain view of organizational learning and the nature of a learning organization. Örtenblad (2004, 2018) has proposed four lenses through which a learning organization can be understood. Our results are especially in line with three of the visions put forward. The first, the organization is understood as a facilitator of learning, where learning emerges while work is done (this process being dubbed “learning at work”), instead of employees being sent to attend formal courses outside the workplace. In our data analysis and the ECSO-Learn model, we suggest that local learning agents should be recruited from their local units; this enables the learning process to take place while work is being carried out, with the learning agent as a learning facilitator. In our model, this consideration is linked to the individual learning process in operation within a learning organization: in particular, the manager and learning agent need to clarify which roles each serves in this process, covering the facilitation of individual learning, as well as any necessary social and emotional support in the process.

In a second vision of the learning organization, Örtenblad (2018) puts forward the view that a learning organization is an organization with a *climate for learning*, in which organizations facilitate learning processes by making room for trial and error. This issue is linked to our group of factors that relate to the learning culture: specifically, the optimal learning culture is one in which the manager, learning agent, and organizational members all contribute to the facilitation of collective learning, engage in critical thinking, and promote collective sharing.

Under the third vision, a learning organization focuses on facilitating *organizational* learning (as opposed to merely *individual* learning). In our analysis and the ECSO-Learn model, this factor is represented by the learning culture as well as the continuous, circular learning process in which influence occurs in all directions between the learning culture, the manager, the learning agent, individual employees, and the desired outcome. From this perspective, organizational memory is upheld through this continuous learning cycle and the learning culture is supported through collective learning, sharing, and critical thinking.

These results, and the proposed model, are in line with the collective, relational, situated, and emerging aspects of organization and leadership (Lave and Wenger, 1991; Barker, 1997, 2001; Mintzberg, 2004; Alvesson and Spicer, 2012; Tourish, 2013), within which an individual (either a manager or a super user or learning agent) should be viewed as part of a collective set of processes. Furthermore, the results are also in line with previous descriptions of learning processes that occur among experienced leaders during their participation in university degree programs, which have underlined the importance of collaborative activities in learning (Rennemo and Vaag, 2018). The results also relate to the integrative approach put forward by Chiva and Alegre (2005).

### Super Users, Change Agents, and Learning Agents

Lunenburg (2010) presents an overview of the role of the change agent based on a literature review, where such an agent is defined as one who has the skills and power to stimulate, facilitate, and coordinate change. This overview includes a list of characteristics for “successful change agentry”:

1. *Homophily* and *Proximity*: The change agent needs to have some similarity to other employees and be close to the other members of the organization, which is linked to our finding that the learning agent should be available to and recruited from the local unit, with adequate local knowledge of the employees and culture.
2. *Empathy*: The agent should have an understanding of employees’ emotions, which is linked to our finding of the need to provide emotional support in the individual learning process.
3. *Capacity*, *Linkage*, and *Openness*: The organization needs to be capable of providing resources for change and engaging its members in collaborative activities, as well as being open to others’ opinions and influence; this is linked to our results underlining the need for facilitation of a learning culture through collective learning, sharing, and critical thinking.
4. *Structuring*: The need for planning and structuring of change could be linked to our results indicating that the manager’s and learning agent’s roles should be continuously negotiated throughout the process of a change and/or implementation initiative.

### Selection of a Learning Agent (Super User)

To address the need for learning to emerge in the process of work, to facilitate a culture of learning, and to maintain a continuous learning process, our results show that a learning agent targeting a local unit should be recruited from that unit. In addition to possessing local knowledge and fundamental technological skills, the agent should have the pedagogical skills required to facilitate learning and to contribute positively to the learning culture, which should also include their proactive involvement in the learning process. Through dialogue with the local manager, the learning agent ideally clarifies their roles and responsibilities to contribute to the circular learning process. It will be easier to recruit learning agents to facilitate learning processes on both the organizational and the individual levels in an organization with a mature learning culture. In the context of such a culture, it would also be reasonable to think that the learning agent could, in broader terms, represent the members of the organization in cases where simultaneous learning processes are taking place and different learning agents take on different responsibilities.

### Implications and Further Research

This research topic is of importance in understanding how organizations can best maintain productivity during the implementation of new technology and optimize this implementation phase itself. The introduction of new technology is a complete routine but also an important occurrence in modern organizational life, and a successful implementation phase is crucial. A typical organization is likely to implement new technology regularly, and our research provides an understanding of the optimal process involved in accomplishing this.

Our project involved a major technological implementation in a Norwegian municipality within the context of the Norwegian organization model. An investigation of the understanding and use of super users should also be conducted within other contexts. We did also choose to use informants in this project that were more involved with the use of the technology daily. Thus, although the informants are from different units, the informants may consider themselves to be from the same community of practice (Lave, 1991; Wenger, 1998). Our organization at hand could also be described as a fairly stable organization, with a less degree of turnover than one could expect. In cases where the new technology is less a part of the daily working routine and tasks (or less a part of their domain), and/or the members of the unit are less fixed, we probably could expect it to be more of a challenge to recruit members from the learning culture (or the community of practice) to be super users. Conceptually, the ECSO-Learn model should be critically investigated and linked to existing models of organizational learning. This should involve research employing both qualitative and quantitative methodologies, investigating how it is applicable when the technology is less a part of the daily routine. More qualitative and observational research is needed to understand the specific components and processes involved in the provision of learning at the organizational and individual level, involving a larger variety of informants than in this project, from different contexts.

A survey based on the model should be designed, validated, and tested to promote organizational learning and to determine the most relevant and important aspects of the learning agent’s roles and responsibilities. To investigate the effects of the use of super users and/or learning agents, intervention studies are also needed; this work should involve expertise from both the technological and the social sciences.

## Conclusion

This study investigated the criteria that should be used in the selection of a super user and the allocation of responsibilities between the roles of super users and managers in an organization that is implementing the introduction of new technology. Our results indicate that super users should be recruited from their local unit, and that *learning agent* could be a better label than super user for this role, indicating the need for skills that go beyond technological competence. Pedagogical competence and an engaged, proactive, and collaborative attitude are also prominent in the descriptions of the skills required for learning agents (super users). We also found that learning processes related to the introduction of new technology should not be separated from the more general continued organizational learning processes that are in operation in an organization. The learning agent and manager should each be engaged in the process of facilitating learning on the organizational, cultural, and individual levels to facilitate long-term organizational learning processes, in a pattern described by the model that we have termed the ECSO.

In layperson’s terms, the super user should not be a technological Superman recruited from the planet Krypton, but rather a Clark Kent recruited from next door, with insight into both the new technology and the uniqueness of the organization, as well as the pedagogical and collaborative skills needed to facilitate learning.

## Data Availability Statement

The data presented in this article are not readily available because data are based on qualitative interviews that may be subject to indirect personal identification. We do not have ethics approval for sharing of such data. We can provide additional quotes that correspond to the themes described in results. Requests to access this should be directed to JV, jonas.vaag@nord.no.

## Ethics Statement

The studies involving human participants were reviewed and approved by the Norwegian Centre for Research Data. The patients/participants provided their written informed consent to participate in this study.

## Author Contributions

JV, TH, and SS planned and administered the project. TH and SS developed the interview guide, recruited informants and conducted interviews under guidance of JV. TH, SS, and JV did the initial analysis, with contributions from GS. TH, SS, GS, and JV drafted the initial manuscript and worked together in forming the final manuscript. All authors contributed to the article and approved the submitted version.

## Conflict of Interest

The authors declare that the research was conducted in the absence of any commercial or financial relationships that could be construed as a potential conflict of interest.

## Publisher’s Note

All claims expressed in this article are solely those of the authors and do not necessarily represent those of their affiliated organizations, or those of the publisher, the editors and the reviewers. Any product that may be evaluated in this article, or claim that may be made by its manufacturer, is not guaranteed or endorsed by the publisher.
